# Isolated Lung Ventilation With Tracheostomy Using Two Intubation Tubes for Severe Endotracheal Hemorrhage Due to Pulmonary Contusion

**DOI:** 10.7759/cureus.64443

**Published:** 2024-07-13

**Authors:** Hiroshi Matsuura, Yuki Matsui, Kazunori Okuda, Masafumi Kishimoto

**Affiliations:** 1 Emergency and Critical Care, Osaka Prefectural Nakakawachi Emergency and Critical Care Center, Osaka, JPN

**Keywords:** acute trauma care, tracheostomy, intubation tube, pulmonary contusion, isolated lung ventilation

## Abstract

A 72-year-old man presented with severe pulmonary contusions and multiple traumas, including aortic injury, pelvic fracture, and renal injury. The patient required multidisciplinary treatment, including transcatheter arterial embolization, thoracic endovascular aortic repair, right lung upper lobe partial resection, and massive transfusion. During the initial treatment, the patient experienced respiratory failure due to endotracheal bleeding, and we attempted isolated lung ventilation with a 37 Fr double-lumen endotracheal intubation tube. Although drainage by suction and protection of the healthy lung was vital, the patient was unable to maintain ventilation volume because of poor drainage. Additionally, the respiratory status deteriorated. To resolve the situation, a tracheotomy was performed and two endotracheal intubation tubes (6.0 mm inner diameter, and 9.0 mm outer diameter) were inserted through a large U-shaped tracheal hole 18 hours after admission. The respiratory status of the patient gradually improved after the procedure. There were two advantages of this method of respiratory management. Firstly, each of the two endotracheal tubes had a separate cuff, allowing more reliable separation of the healthy lung from the injured lung. Secondly, bronchoscopes of sufficient diameter (4.9 mm outer diameter ) were used bilaterally, allowing sufficient drainage of viscous airway secretions mixed with hematoma and improving atelectasis. Although venovenous extracorporeal membrane oxygenation is a crucial support tool when the respiratory status deteriorates due to severe pulmonary contusions, our method of airway management may be attempted in patients with multiple traumatic injuries with coagulopathy.

## Introduction

Severe pulmonary contusions cause endotracheal hemorrhage, resulting in poor ventilation and respiratory failure. Furthermore, in cases of multiple traumas, other injuries are combined with severe pulmonary contusions, and coagulopathy may be impaired, making it challenging to control the hemorrhage.

Independent lung ventilation is the primary intervention and rescue ventilation strategy for both anatomical and physiological lung separation [1]. Although reports indicate the use of isolated lung ventilation with double-lumen tracheal tubes and intubation tubes [2,3], it is challenging to use a bronchoscope of an appropriate diameter with a double-lumen tube in situations where sufficient drainage is needed in both lungs. Herein, we report a novel case of isolated lung ventilation with tracheostomy using two endotracheal tubes in the acute phase to manage respiratory failure in severe pulmonary contusions.

## Case presentation

**Figure 1:**
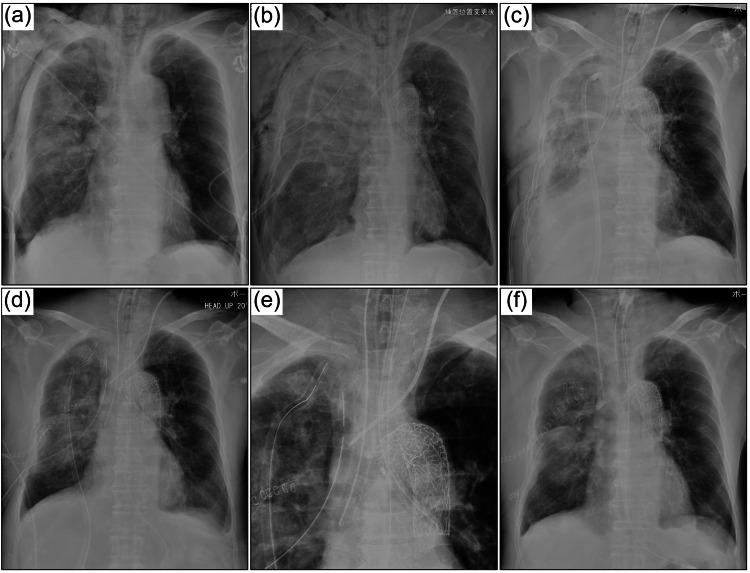
Serial change of chest XP. (a) Admission. (b) After thoracic endovascular aortic repair (TEVAR) and lung resection. (c) Immediately after tracheostomy. (d) Day 2. (e) Expansion of intubation tube location on Day 2. (f) Day 8 (The tracheostomy tube was replaced with a regular tube). XP: X-ray photograph.

A 72-year-old man sustained an injury after falling from a height of 3 m while working. On arrival at the hospital, the blood pressure of the patient was 105/78 mmHg, heart rate (HR) 139/min, respiratory rate 31/min, oxygen saturation (SpO_2_) could not be measured, and body temperature was 35.8℃. Chest radiography (Figure 1a) revealed pneumothorax and pulmonary contusion, and the patient had respiratory failure.

**Figure 2:**
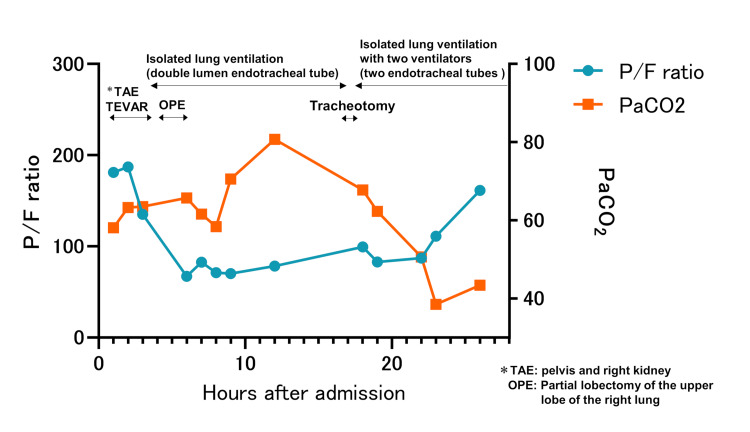
Clinical course of the patient on Day 1. After tracheostomy, respiratory status improved markedly with good bronchoscopic drainage. TAE: Transcatheter Arterial Embolization, OPE: Operation, TEVAR: Thoracic endovascular aortic repair, PaCO_2_: Partial pressure of carbon dioxide.

**Figure 3:**
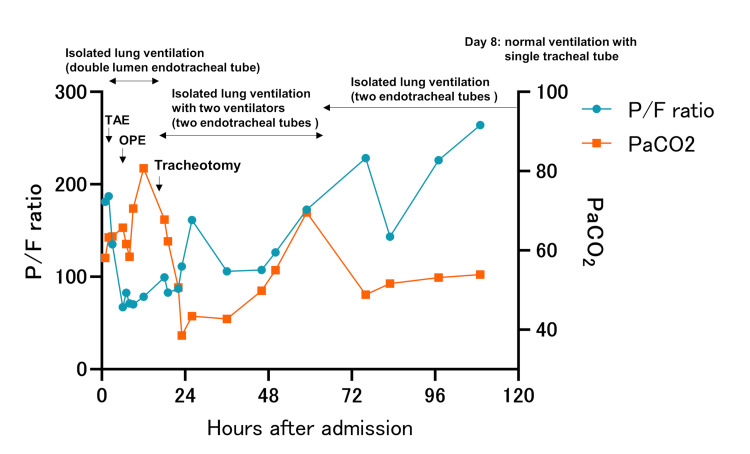
Clinical course of the patient five days after admission. Sixty hours after the patient's arrival, the ventilator was consolidated to one. TAE: Transcatheter arterial embolization, OPE: Operation.

Enhanced computed tomography (CT) revealed a severe pulmonary contusion on the right side with venous hemorrhage, multiple rib fractures, aortic injury distal to the aortic arch, right renal injury, and a pelvic fracture. Transcatheter arterial embolization (TAE) was first performed selectively on the right iliolumbar artery in the pelvis, and on the main trunk of the right internal iliac artery using a gelatin sponge. Subsequently, we performed TAE for the right kidney injury using a gelatin sponge and coil, and thoracic endovascular aortic repair (TEVAR) for the aortic arch injury. During TEVAR, the patient developed progressively worsening respiratory function, including hypoxia and carbon dioxide retention, which were attributed to the bleeding from a pulmonary contusion. After the interventional radiology, preoperatively, the endotracheal tube of the patient was switched to a 37 Fr double-lumen intubation tube (Broncho-Cath^TM^; Covidien Japan Inc., Tokyo, Japan) with isolated lung ventilation, and was subsequently operated in the left lateral position. In emergency situation, the maximum size of a double lumen tube size is 37 Fr at our hospital. Intraoperative findings revealed continued bleeding from a pulmonary contusion in the right upper lobe. The contusion site was resected using an automatic suture. Furthermore, the patient experienced bleeding from a rib fracture on the chest wall, which was ligated to terminate the bleeding. Postoperatively, the patient was placed in the intensive care unit (ICU) with continuous isolated lung ventilation using a Broncho-Cath^TM^ to protect the healthy lung from the injured lung. Red blood cell concentrate (3920 ml), freshly frozen plasma (4560 ml), and platelet concentrate (500 ml) were transfused before admission to the ICU. Although drainage by suction and protection of the healthy lung was vital, the patient was unable to maintain ventilation volume because of poor drainage. Subsequently, the respiratory status deteriorated (Figures 2, 3).

**Figure 4:**
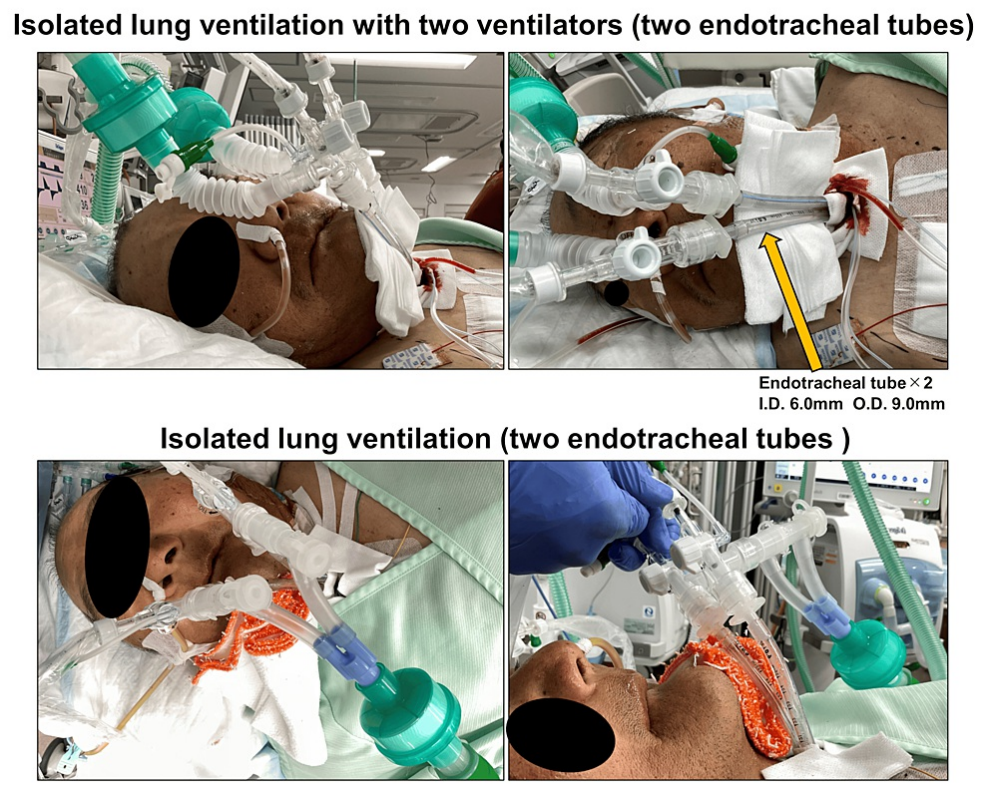
Isolated lung ventilation with two ventilators (two endotracheal tubes). A tracheotomy was performed and two endotracheal intubation tubes with suction holes over the cuff (ID 6.0 mm and OD 9.0 mm) were inserted through a large U-shaped tracheal hole 18 hours after the patient's admission. ID: Inner diameter, OD: Outer diameter.

This was attributed to airway obstruction due to bleeding from the residual contused lung, and a sufficient bronchoscope (4.9 mm outer diameter {OD}) could not be used on either side with the current double-lumen endotracheal tube. As bleeding was under control and ventilation and drainage problems required resolution, a tracheotomy was performed, and two endotracheal intubation tubes with a suction hole over the cuff (6.0 mm inner diameter {ID} and 9.0 mm OD) were inserted through a large U-shaped tracheal hole after 18 hours of admission (Figure 4).

Using a bronchoscope, the tip of the left tube was positioned slightly proximal to the upper lobe branch, and the right tracheal tube was fixed in a position where the upper lobe branch was visible through the side hole of the tube. A ventilator was used for each tracheal tube, and the left lung (healthy side) was ventilated with bilevel positive airway pressure (BIPAP) mode, the fraction of inspired oxygen (FiO_2_) at 80%, peak inspiratory pressure (PIP) 28 cmH2O, positive end-expiratory pressure (PEEP) 12 cmH2O, and respiratory rate 16 beats/min. The right side (injured side) was ventilated with continuous positive airway pressure (CPAP) mode, FiO_2_ at 80%, and PEEP 12 cmH2O with no ventilation under deep sedation, including muscle relaxation. After the tracheostomy, the respiratory status improved significantly with good bronchoscopic drainage. Sixty hours after the arrival of the patient, the ventilator was consolidated to one (Figure 3). The tracheostomy tube was replaced with a regular tube on the 8th day (Figure 1f). The patient improved to the point where he was able to stand and tolerate oral intake. The patient received only 3 L/min of oxygen and was subsequently transferred to the hospital for further rehabilitation on the 34th day.

## Discussion

Severe pulmonary contusions are associated with high mortality rates and require multidisciplinary treatment. Although venovenous extracorporeal membrane oxygenation (V-V ECMO) improves the survival of adult trauma patients with respiratory failure [4], bleeding has been associated with higher mortality [5]. Bleeding occurs in 21-66% of patients on V-V ECMO [5,6]. Furthermore, gastrointestinal bleeding, hemothorax, and retroperitoneal hematomas may cause hemorrhagic shock [6]. The risk may be higher in patients with severe pulmonary contusions and multiple traumas with possible coagulopathy. Hence, V-V ECMO should be considered only if it cannot be avoided.

The OD of the suction tube attached to the 37 Fr endotracheal double lumen intubation tube and tracheal tube for isolated lung ventilation was 10 Fr (3.3 mm OD); however, only a narrow diameter scope (3.1 mm OD; Evis Lucera Elite Broncho video scope BF-XP290, Olympus Corporation, Tokyo, Japan) could be used. The size of the patient's endotracheal lumen was 18mm, thus 39 Fr endotracheal double lumen tube is better for using a suitable bronchoscope (4.9 mm OD; Evis Lucera Broncho video scope BF 260, Olympus Corporation, Tokyo, Japan). However, in an emergency situation, the maximum size of the double lumen tube is 37 Fr at our hospital. In this case, a suitable bronchoscope (4.9 mm OD) that would allow adequate drainage was not available, forming atelectasis and resulting in respiratory failure.

Herein, we report the rare case of a patient with multiple traumas, in whom a modified airway management strategy saved the life of the patient without V-V ECMO. The advantages of management with isolated lung ventilation through two-endotracheal tube tracheotomy are as follows: i) Each of the two endotracheal tubes has its cuff, allowing a more reliable separation of the healthy lung from the injured lung, and each cuff blocks aspiration and a suction hole over the cuff allow good drainage; ii) Bronchoscopes of sufficient diameter (4.9 mm OD) can be used bilaterally, allowing sufficient drainage of viscous airway secretions mixed with hematoma and improving atelectasis.

Several points should be noted regarding the implementation of this procedure. The left side should be fixed in a position where the upper lobe of the left bronchus can be visualized. The right side requires some ingenuity because anatomical challenges facilitate the formation of atelectasis in the upper lobe.

On the right side, it is vital to position the lateral hole of the endotracheal tube in line with the bifurcation of the right upper lobe, which can be confirmed by visualizing the right upper lobe through the lateral hole using a bronchoscope. This requires not only matching the insertion length but also rotating the tube to the appropriate position.

## Conclusions

Isolated lung ventilation was performed using two endotracheal intubation tubes through a tracheostomy hole in a patient with a severe pulmonary contusion. Although venovenous extracorporeal membrane oxygenation (V-V ECMO) is a vital support tool when the respiratory status deteriorates due to severe pulmonary contusions, our method of airway management may be attempted in patients with multiple traumatic injuries with coagulopathy.
